# Innovative method for Amplatzer device implantation in patients with bronchopleural fistulas

**DOI:** 10.1186/s12890-021-01493-8

**Published:** 2021-04-26

**Authors:** Jisong Zhang, Huihui Hu, Li Xu, Shan Xu, Jihong Zhu, Fengjie Wu, Enguo Chen

**Affiliations:** 1 Department of Pulmonary and Critical Care Medicine, Sir Run Run Shaw Hospital of Zhejiang University, No. 3 East Qingchun Road, Jianggan District, Hangzhou, 310016 Zhejiang Province China; 2 Department of Anesthesiology, Sir Run Run Shaw Hospital of Zhejiang University, Hangzhou, Zhejiang Province China; 3 Department of Pulmonary and Critical Care Medicine, The Second Hospital of Jiaxing, Jiaxing, Zhejiang Province China

**Keywords:** Bronchopleural fistula, Amplatzer devices, Pulmonary intervention, Bronchoscopy, Sheath-free method

## Abstract

**Background:**

Bronchopleural fistula (BPF) is a relatively rare complication after various types of pulmonary resection. The double-sided mushroom-shaped occluder (Amplatzer device, AD) has been gradually used for BPF blocking due to its reliable blocking effect. We have improved the existing AD implantation methods to facilitate clinical use and named the new approach Sheath-free method (SFM). The aim of the present report was to explore the reliability and advantages of the SFM in AD implantation.

**Methods:**

We improved the existing implantation methods by abandoning the sheath of the AD and using the working channel of the bronchoscope to directly store or release the AD without general anesthesia, rigid bronchoscopy, fluoroscopy, or bronchography. A total of 6 patients (5 men and 1 woman, aged 66.67 ± 6.19 years [mean ± SD]) had BPF blocking and underwent the SFM in AD implantation.

**Results:**

AD implantation was successfully performed in all 6 patients with the SFM, 4 persons had a successful closure of the fistula, one person died after few days and one person did not have a successful closure of the fistula. The average duration of operation was 16.17 min (16.17 ± 4.67 min [mean ± SD]). No patients died due to operation complications or BPF recurrence. The average follow-up time was 13.2 months (range 10–17 months).

**Conclusion:**

We observed that the SFM for AD implantation—with accurate device positioning and a clear field of vision—is efficient and convenient. The AD is effective in BPF blocking, and could contribute to significantly improved symptoms of patients.

**Supplementary Information:**

The online version contains supplementary material available at 10.1186/s12890-021-01493-8.

## Background

Bronchopleural fistula (BPF) is a serious complication that occurs after various types of pulmonary resection. The incidence of BPF following surgery is 4.4–8.0% [1, 2], and BPF places a substantial economic and spiritual burden on patients [3, 4]. It likely results from preoperative neoadjuvant chemotherapy, operations on the right side, and complete pneumonectomy [5]. Once appears, it often causes challenging management problems with the mortality rate ranging from 18 to 50% [3, 4].

BPF is handled with comprehensive treatment, including closed thoracic drainage, prolonged antibiotic use, symptomatic supportive treatment, and various fistula blocking methods [6]. Several studies found that blocking BPF by means of respiratory endoscopy has the advantages of high patient acceptance, low operation risks, low overall costs, and rapid postoperative recovery [7, 8]. Endoscopic interventional treatment for BPF currently utilizes two major methods: one is to stimulate the formation of local granulation tissue and scar tissue through various kinds of physical and chemical methods to achieve a blocking effect; the other is to place various types of occluders, including distally closed metal stents, distally closed silicone stents, EBVs (Endobronchial Valves), and Amplatzer devices [9–12].

Fruchter O first reported the use of double-sided mushroom umbrella occluders (Amplatzer devices, ADs) or arterial catheter occlusion devices (Amplatzer vascular plugs, AVPs) in treating BPFs [13–15]. Different methods for AD implantation have been reported in previous studies. According to Fruchter O, ADs are implanted under direct bronchoscopic and fluoroscopic visualization with the use of guide wires passed through the fistula as aids [15, 16]. In China, the common method of implantation is rigid bronchoscopy, or via tracheal intubation with the guidance of bronchoscopy passed through the nasal passage. However, almost all of the above methods require general anesthesia, time-consuming and troublesome, and there is a possibility of implantation failure. In the current study, we described a novel and innovative method (Sheath-free method, SFM) for AD implantation which may make AD implantation more convenient and efficient in clinical use.

## Methods

**Fig. 1:**
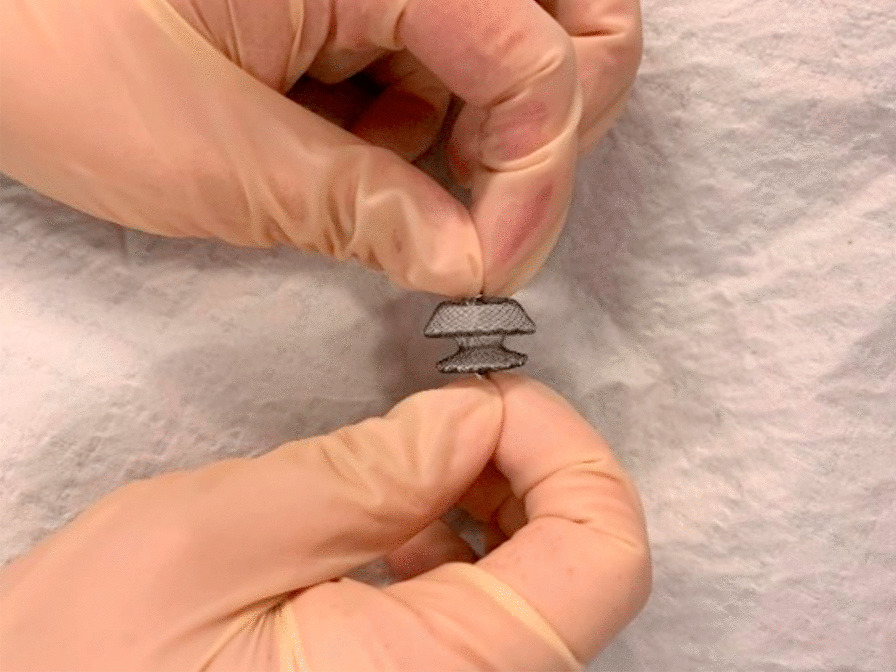
ADs used for bronchoscopic closure of bronchopleural fistulas

ADs (Fig. 1) are self-expanding double-sided mushroom umbrella structures woven from nickel-titanium alloy wires with a slender waist in the middle. In this study, we used the ADs ordered from VISEE medical Co. (Shandong, China) for study. Their sealing disc diameters range from 12 to 56 mm, and waist diameters range from 4 to 38 mm. Here, ADs with waist diameters between 6 and 12 mm were selected. The specific procedure was carried out at the bronchoscopy operating room in patients under moderate sedation, unless they were already mechanically ventilated. Totally 6 patients were included and all of them received topical anesthesia, with lidocaine, dextromethorphan and remifentanil continuously administered for maintenance. No general anesthesia was taken. After sedation, bronchoscopy was used to observe fistulas and the suitable AD model was selected. The fistulas of all the 6 patients were visible under the direct vision of the bronchoscope, with no need for additional means such as bronchography for determination.

**Fig. 2:**
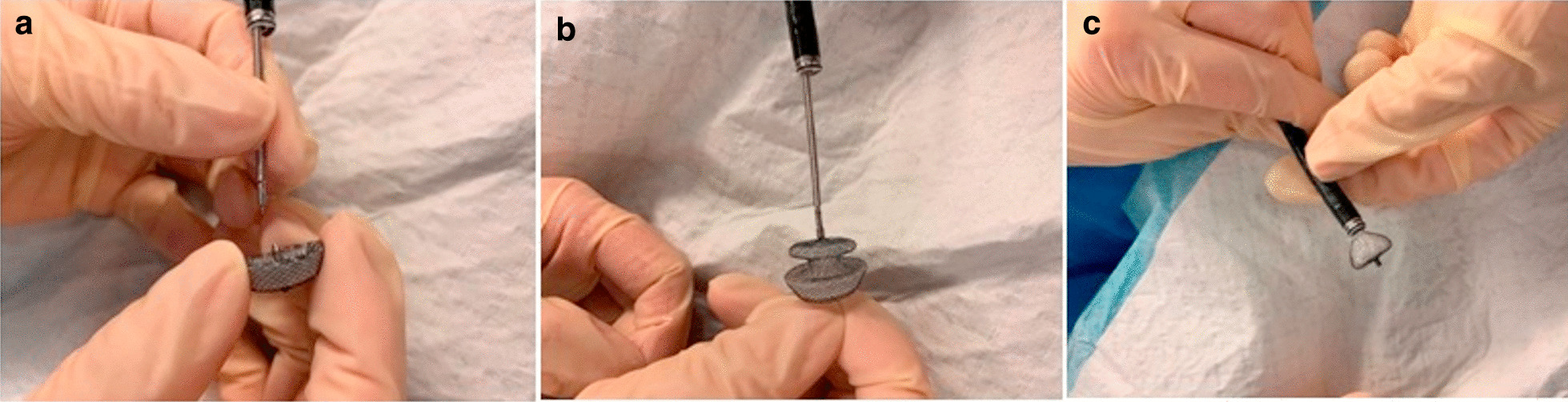
Procedures of the Sheath-free method. **a** Guide wire entry through the working channel. **b** AD connected to the guide wire. **c** AD integrated into the working channel

The innovation of SFM is the replacement of the sheath tube of the AD by a bronchoscopy working channel. An Olympus T series endoscopy (model Bf-it290, outer diameter of 5.9 mm and a working channel of 2.8 mm, Olympus Corp, Tokyo, Japan) was selected here for implantation. A guide wire (diameter of 1.9 mm) was inserted into the working channel of the bronchoscope. After it extended out of the working channel, it was connected to the AD, so that the AD could be received or released by drawing the guide wire. The operation process is shown in Additional file 1: Video S1. The release process was performed directly under the bronchoscope with a clear field of vision, and the AD could be adjusted by drawing the guide wire at any time until it was satisfactory. Upon reaching a satisfactory position, the device was detached. The bronchoscope was removed from the airway, and the patient was transferred to the recovery room. The device can be placed through the nasal passage, a tracheal tube, a laryngeal mask, or a rigid bronchoscopy, depending on the patient's personal conditions. It is very difficult to remove the AD after implantation for its special double-sided mushroom umbrella structure, and it is often removed by surgery. The procedures are presented in Fig. 2. Before AD implantation, we reported to the hospital ethics committee for approval of the study, and all the patients included were informed of the study procedures and signed informed consent as well as off-label use consent forms.

## Results

In total, 6 patients (5 men and 1 woman, aged 66.67 ± 6.19 years [mean ± SD]) were treated in our center with AD implantation by SFM under bronchoscopy between October 2018 and May 2019. The device was successfully implanted in 6 persons, 4 persons had a successful closure of the fistula, one person died after few days and one person did not have a successful closure of the fistula. After occlusion surgery, all the patients, except patient No. 6, achieved complete occlusion, and symptoms related to BPF disappeared following AD blocking. Three out of the 5 patients with complete occlusion were then free of the thoracic drainage tube.

As observed, pneumonectomy (n = 3) and lobectomy (n = 3) were the main etiologies for BPF, and primary lung cancer (n = 6) turned out to be the underlying disease in all patients. The demographic and treatment data for the study participants are presented in Tables 1 and 2. The average follow-up time for all patients was 13.2 months (range: 10–17 months). All the 6 patients underwent AD implantation with the use of the SFM, and the average duration of operation was 16.17 min (16.17 ± 4.67 min [mean ± SD]).

**Table 1:** Patient characteristics and treatment data

Serial number	Operative site	Disease	Onset time of BPF	Location of BPF	Indwelling time of drainage tube
1	Lower right lobectomy	Adenocarcinoma	8 months after operation	Right lower bronchus	2 months
2	Lower right lobectomy	Squamous cell carcinoma	20 days after operation	Right lower bronchus	15 days
3	Left pneumonectomy	Squamous cell carcinoma	9 days after operation	Left main bronchus	2 months
4	Upper left lobectomy	Squamous cell carcinoma	40 days after operation	Upper left bronchus	20 days
5	Right pneumonectomy	Non-small-cell lung cancer	1–2 months after operation^a^	Right main bronchus	23 years
6	Right middle lobe and right lower lobe lobectomy	Squamous cell carcinoma	1 month after operation	Right middle and lower bronchi	2 months

^a^The patient has been experiencing BPF for 23 years, hence it is difficult for him to remember the exact time

**Table 2:** Operation and follow-up information of 6 patients

Serial number	Location of BPF	Fistula diameter	AD model	Duration of operation	Completely blocked	Removed drainage tube	Time from closure to extubation	Follow-up time
1	Right lower bronchus	10 mm	22-10-24 mm	10 min	Yes	Yes	3 days	17 months
2	Right lower bronchus	8 mm	16-8-20 mm	15 min	Yes	Yes	3 months	15 months
3	Left main bronchus	10 mm	23-12-27 mm	18 min	Yes	No	NA	NA
4	Upper left bronchus	8 mm	16-8-20 mm	20 min	Yes	Yes	2 months	12 months
5	Right main bronchus	10 mm	22-10-24 mm	22 min	Yes	Follow-up	NA	12 months
6	Right middle and lower bronchi	Several small fistulas	23-12-27 mm	12 min	No	Yes^b^	NA^b^	10 months

^a^Although patient No. 3 underwent closure in the ICU with respiratory failure was observed to have a significant reduction in air leakage after AD implantation, he eventually died on the third postoperative day due to complications from severe pneumonia

^b^Patient No. 6 underwent thoracoscopic flap sealing after closure failure of AD implantation, and his drainage tube was removed one week after surgery

AD implantation was successfully performed in all 6 patients. There were no immediate complications related to the procedure, and all patients were discharged within 24 h, except patient No. 3 who underwent closure in the ICU (intensive care unit) and had severe pneumonia of the residual right lung. Although this patient was observed to have a significant reduction in air leakage after AD implantation, he eventually died on postoperative day 3 due to complications of severe pneumonia. Patient No. 6 whose bronchoscopy showed multiple micro fistulas at the end of the right middle bronchus received AD closure, but we observed persistent air leakage in the water seal drainage bottle at 6 months after AD implantation. Finally, the patient underwent thoracoscopic free anterolateral thigh flap sealing, and his drainage tube was removed one week after surgery. No patient died from operation complications or BPF recurrence.

**Fig. 3:**
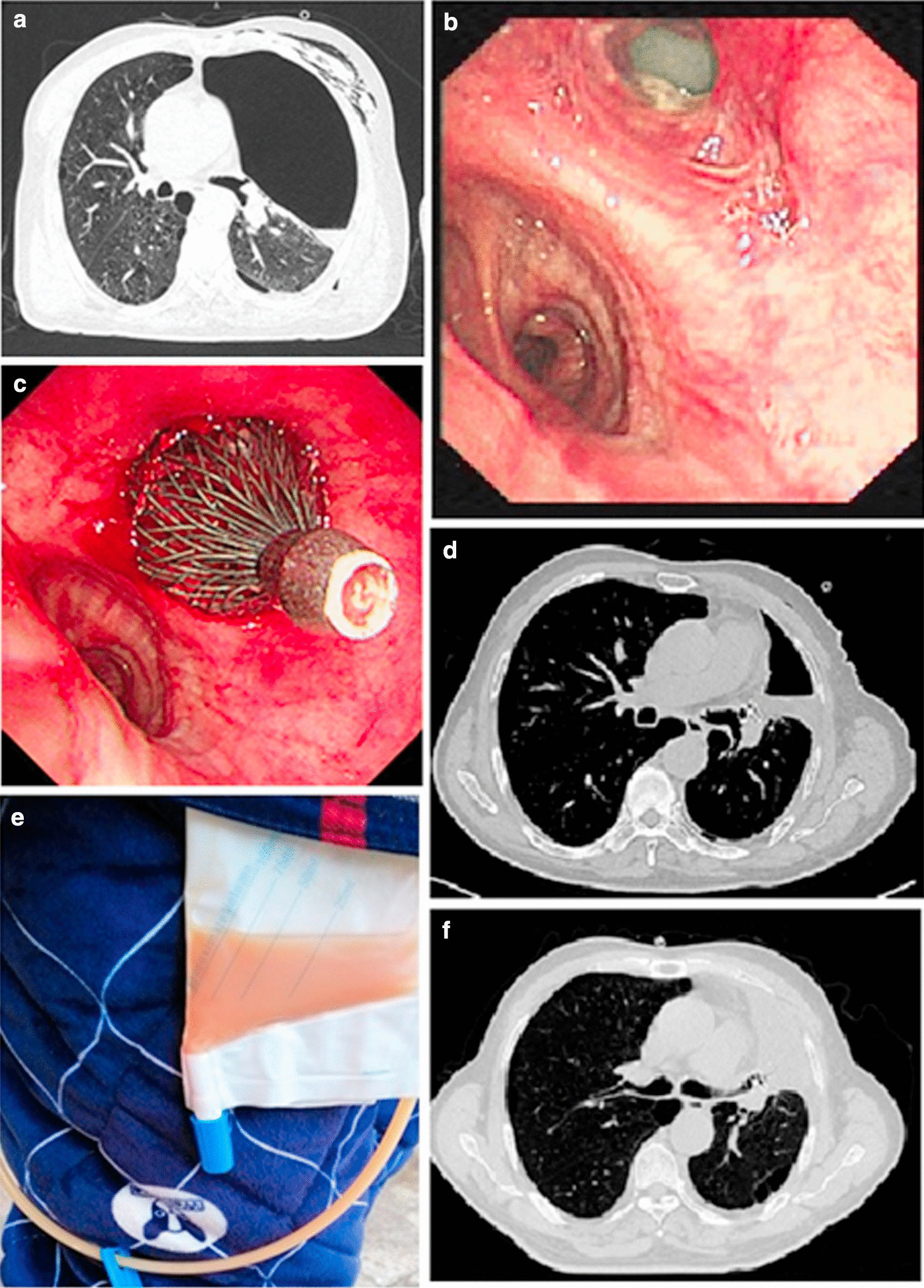
Bronchopleural fistula following left upper lobe lobectomy in patient No. 4. **a** Chest CT before AD implantation. **b** Bronchoscopy before AD implantation. **c** Bronchoscopy after AD implantation. **d** Chest CT after 1 month of AD implantation. **e** Water seal drainage bottle was changed to a drainage pack after 1 month of AD implantation. **f** Chest CT after 2 months of AD implantation, and the drainage tube was removed after CT scan

**Fig. 4:**
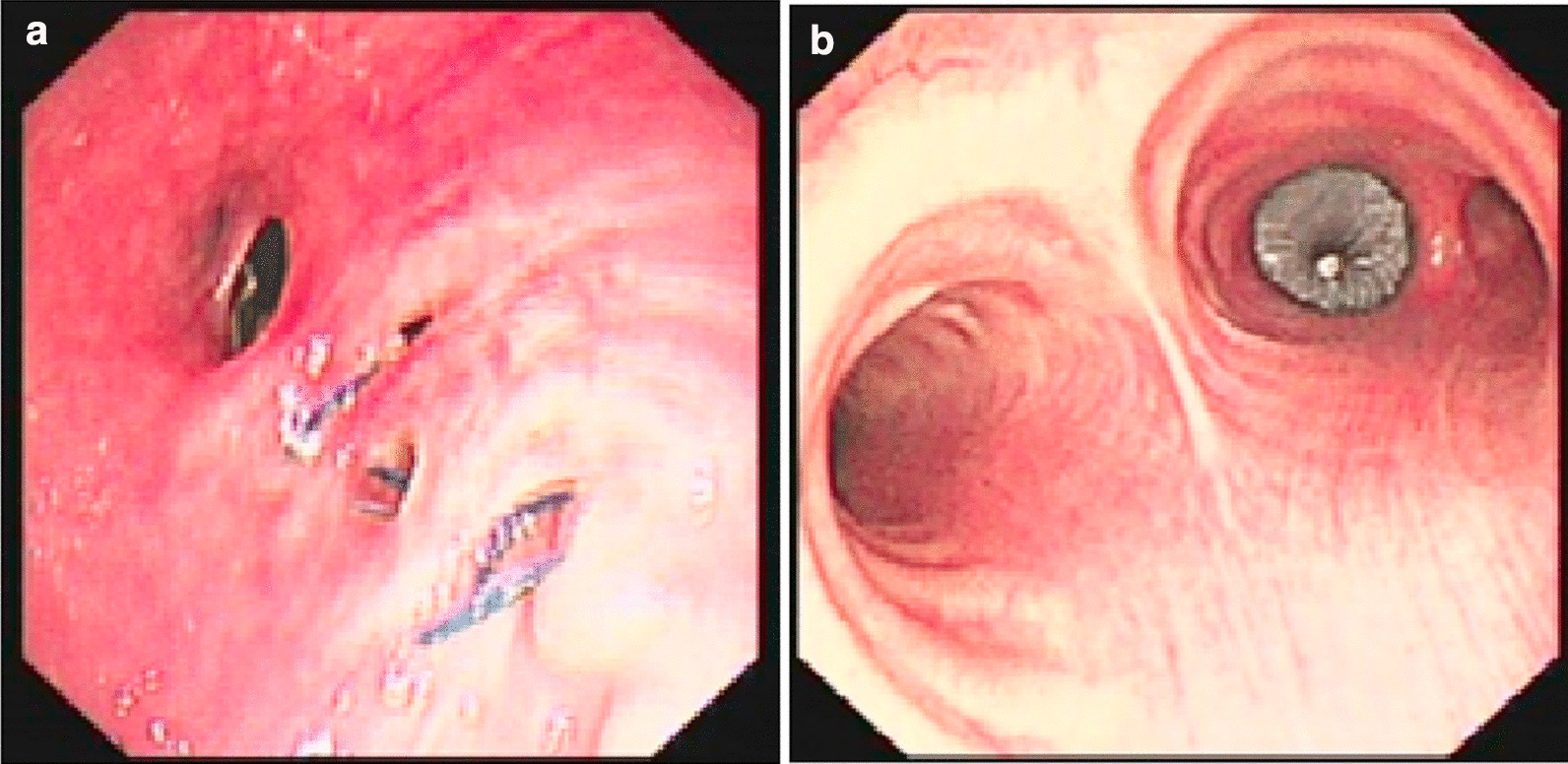
Bronchopleural fistula following right middle lobe and right lower lobe lobectomy in patient No. 6. **a** Bronchoscopy before AD implantation. **b** Bronchoscopy after AD implantation

Follow-up evidence exhibited a definite blocking effect of the AD implantation and significant improvement of patient’s symptoms. The first improvement after the AD implantation was reduction in phlegm volume, followed by reduced cough symptoms. After 3 months of follow-up, improvements in the overall condition, such as exercise tolerance, weight gain, improved stomach intake, and a more positive attitude, were often observed. Representative figures of the study patients are presented in Figs. 3 and 4.

## Discussion

This study presented an innovative method (SFM) for AD implantation for the first time and indicated its feasibility and advantages in clinical practice, such as easy steps, short operation time, few complications, and ease of reaching the fistula for closure. With this innovative method, AD can be placed through nasal passage, mouth or a laryngeal mask, with no need to use tracheal tube or rigid bronchoscopy, which makes the procedure much easier. This also means that patient under the AD implantation does not need to receive general anesthesia, only topical anesthesia, contributing to the reduction in the cost and duration of operation, the risk of anesthesia, and the incidence of complications. In our study, the shortest duration of the whole implantation operation lasted only 10 min (Patient No. 1 in Table 2). However, no average time for AD implantation was found as a reference with a paucity of data on duration of operation that was previously presented by other scholars. Regardless of this limitation, we speculated that the duration of other operation may be more than 30 min according to the description of operation procedures, such as the need for rigid bronchoscopy or bronchography performance. Tedde ML reported a case with right upper lobe BPF who received AD implantation introduced by sheath which was advanced over the guidewire in the working channel in a 60-min procedure [17]. A shorter duration of operation could reduce the risk of mechanical ventilation and anesthesia, which is conducive to the safety of AD implantation and reduction of complications. Here, postoperative CT showed that the accuracy of the AD implantation with SFM was also favorable (Fig. 2). It is not easy to accurately place an AD into the fistula, especially for fistulas in the upper lobe which are hard to reach. In our study, although patient No. 4 suffered from an upper left BPF, the implantation with SFM was completed by smoothly reaching the fistula, which only took 20 min. A previous study reported a case of failed implantation [13]. In this case, the AD fell into the pleural cavity, and the failure may result from severe infection around the fistula. Among the six patients who completed AD implantation in our center, there was no AD drop or displacement, indicating its reliability. Nevertheless, the SFM also has its disadvantage that the bronchoscopy working channel required should be 2.8 mm or larger so that the folded AD can be received smoothly. If one wants to place an AD with a large size (for example, 25-14-29 mm or the one described above), the diameter of the AD after folding may be greater than 2.8 mm, which is not suitable for SFM. Additionally, different brands of ADs may have variations in size after folding, which would require much attention when operating. Regarding safety, our observations are consistent with previous studies. The technique employed was well tolerated by the patients without severe side effects or complications.

## Conclusion

In general, the application prospect of ADs in BPF patients is quite optimistic due to the unique advantages. Meanwhile, as a minimally invasive and efficient method, the SFM for AD implantation is safe, convenient and worth spreading, while this conclusion will be more convincing via further verification of its effectiveness and safety.

## Supplementary Information

**Additional file 1.** Operation Display video.

## Data Availability

The datasets used and analysed during the current study are available from the corresponding author on reasonable request.

## Abbreviations

BPF: Bronchopleural fistula
AD: Amplatzer device
SFM: Sheath-free method
EBVs: Endobronchial valves
